# Reproductive Investment and Health Costs in Roma Women

**DOI:** 10.3390/ijerph14111337

**Published:** 2017-11-03

**Authors:** Jelena Čvorović, Kathryn Coe

**Affiliations:** 1 Institute of Ethnography, Serbian Academy of Sciences and Arts, 11000 Belgrade, Serbia; 2 Richard M. Fairbanks School of Public Health, Indiana University–Purdue University Indianapolis, Indianapolis, IN 46202-2872, USA; coek@iu.edu

**Keywords:** Roma, women, reproductive investment, health

## Abstract

In this paper, we examine whether variation in reproductive investment affects the health of Roma women using a dataset collected through original anthropological fieldwork among Roma women in Serbia. Data were collected in 2014–2016 in several Roma semi-urban settlements in central Serbia. The sample consisted of 468 Roma women, averaging 44 years of age. We collected demographic data (age, school levels, socioeconomic status), risk behaviors (smoking and alcohol consumption), marital status, and reproductive history variables (the timing of reproduction, the intensity of reproduction, reproductive effort and investment after birth), in addition to self-reported health, height, and weight. Data analyses showed that somatic, short-term costs of reproduction were revealed in this population, while evolutionary, long-term costs were unobservable—contrariwise, Roma women in poor health contributed more to the gene pool of the next generation than their healthy counterparts. Our findings appear to be consistent with simple trade-off models that suggest inverse relationships between reproductive effort and health. Thus, personal sacrifice—poor health as an outcome—seems crucial for greater reproductive success.

## 1. Introduction

Despite the fact that reproduction and childrearing appear central to the lives of many Roma women, little is known about any effect that reproduction might have on their health. In this paper, we address this gap in knowledge using a dataset collected through original anthropological fieldwork conducted among Roma women living in Serbia.

The Roma, a diverse population of South Asian stock, have been living in Europe for centuries but their integration is poor. For the most part, they remain confined to segregated communities, characterized by poverty, unemployment, poor education, and welfare dependency. Prior studies have shown that Roma communities across Europe tend to have poorer health than the majority population [1,2]. The Roma high birth and mortality rates, impaired health, and shorter life expectancy are usually explained by claiming that they are the consequence of their poverty, low level of education, and socioeconomic status, in addition to inadequate health care and coverage.

A successful reproductive strategy is not without cost; evolutionary life history predicts that the energy invested in reproduction is traded off against investments in maintenance and survival. Thus, having more offspring is associated with the aging process, which may potentially shape other life-history traits [3]. From an evolutionary perspective, this cost has to result in a lower contribution to the gene pool of the next generation, as a consequence of reduced longevity and reduced reproductive success. Individual health is not the anticipated outcome of selection apart from its contribution to reproductive success: When health and reproductive success conflict, selection will favor reproduction at the expense of health [4].

Many studies indicate a trade-off between reproduction, longevity, and health [5,6,7,8,9], but whether or not a (an evolutionary, long-term) cost of reproduction definitively exists among humans is still open to question [10,11,12,13]. In regard to short-term cost, as the direct costs of reproduction—pregnancy, breast-feeding, and childcare—require energy, energetic costs are an essential feature of reproduction [14]. Pregnancy and lactation also involve many physiological changes, including decreases in the functioning of the maternal immune system and increased levels of oxidative stress [15]. The metabolic demands associated with these changes mean that women can experience significant physiological costs associated with reproductive effort and thus are at risk because of the negative consequences of these trade-offs [8].

The trade-off is more critical when the environment does not offer adequate energy [5]. For instance, lifetime parity was demonstrated to shorten the lifespan in some but not all energetically constrained environments [16,17,18,19]. Studies of modern populations have reported a U-shaped effect, in which nulliparous and women with more than four children experience the highest mortality [10]. These results, however, may be confounded by the differences in phenotypic quality: healthy women tend to have both a high fertility and a long life, thus any unfavorable effect of parity on longevity is likely masked by unobserved health characteristics [16,20]. Still, even if total reproduction is not consistently associated with maternal depletion, the timing and intensity of reproduction can still produce somatic costs that can affect different aspects of maternal health and fitness [13].

Studies of the potential costs of reproduction and the effects it may have on health among Roma women are nonexistent despite their lives being characterized by repeated pregnancies, intensive breastfeeding, and many dependent children. To address this gap in knowledge, we conducted a study among low income Serbian Roma, a “hard-to-reach” traditional population. We examined the effect of all measures of reproductive investment, such as timing, the intensity of reproduction, reproductive effort, and investment after birth.

According to official censuses, there are more than 140,000 Roma in Serbia, but the estimates put that number a lot higher at up to 500,000 given the Roma tendency to hide their ethnic origin. Roma women in Serbia, as across much of the world, live in poverty, reside in sub-standard housing in segregated communities, and are not only poorly educated but stigmatized; they lack both the skills for and access to jobs, and have cultural practices that often limit women’s choices [21]. In Serbia, the main characteristics of the female population that declared themselves to be Roma are higher fertility in comparison with non-Roma individuals, which Roma females accomplish in a decade or so: a very high percentage of Roma females had given birth at the beginning of their fertile years (15–24 years), while notably fewer had given birth after the age of twenty five [22,23]. Specifically, 38% of Roma females had their first child before their eighteenth birthday, while 10% became mothers before the age of sixteen. Roma infant and child mortality is estimated to be more than double the national average. Roma children, although coming from large sib-ships, tend not to succumb to nutrition-based mortality risks typical of marginalized populations such as this, but rather most infant and child mortality is due to deaths resulting from neglect, domestic violence, or other unknown or unreported causes [24]. Additionally, among the Roma, the percentage of social care clients is almost four times higher than among the general population in Serbia, which corresponds to the fact that overall and extreme poverty of the Roma population is much higher than for the general population.

## 2. Materials and Methods

The primary aims of this study were to examine the potential costs of reproduction and the effects it has on health among women in several Roma communities in Serbia. Data were collected between 2014 and 2016 in several semi-urban settlements in central Serbia, as part of a larger anthropological study on the health, culture, and social networks among Roma women [25]. The settlements were typical for Roma, with poorly developed infrastructure and poor-quality housing, although there were variations in the local level and also within the settlements. A total of 475 Roma women participated in the study, recruited through personal contacts and Roma organizations; seven women were excluded from the analyses due to missing data. All women had been married at least once, and had given birth to at least one child. 

Informed consent was obtained from all participants and we have observed appropriate ethical guidelines and legislation in conducting the study. Approval to conduct a study of human subjects was awarded by the Indiana University Institutional Review Board (1409034309).

Most women in the sample received social welfare support (70%); only a few women reported that they occasionally work, most often in open markets or as cleaners. All women were fluent in Serbian while some also spoke the Romani language.

A questionnaire was formulated to gather data about Roma women’s demographics (age, school levels, socioeconomic status (SES), lifestyle (risk behaviors such as smoking and alcohol consumption), marital status, and reproductive history (the timing of reproduction: age at first and last reproduction (AFR and ALR); the intensity of reproduction: birth spacing in years; reproductive effort: total number of pregnancies including miscarriages and children that died as infants; and investment after birth: cross-sectional measure of number of surviving children, duration of lifetime breastfeeding in months, and health status of one’s children). In regard to measures of SES, it is difficult to estimate SES for the Roma since, in their case, traditional objective measures of socio-economic status (such as education and income) are not very instructive. In Serbia, the majority of Roma survive through a combination of social benefits and informal work. In the absence of more objective measures, internal Roma women’s own perceptions of (their husband’s) family social standing relative to others in their communities were used instead.

Health status was self-reported (SR). The use of self-reported health as a summary measure of overall health is common in field surveys and independently predicts health outcomes, including all-cause mortality, morbidity, and health service utilization [26,27]. Roma women tend to define health in terms of “normal” everyday functioning and well-being [21]. Nevertheless, among the Roma, as among other socially disadvantaged populations, self-reported health may also reflect shared social experiences, as well as a lack of awareness of asymptomatic diseases [28]. Thus, we asked study participants whether their doctor had told them that they have a chronic disease, the most common being diabetes and hypertension. The responses were 1 for “Yes”, i.e., poor health; and 0 for “No”, i.e., good health. A question on children’s health (coded 1-poor health, coded 0-good health) was included to differentiate children’s characteristics as many studies have found a positive correlation between parent’s and children’s health. Parents of children with chronic health conditions show higher levels of stress which generally correlates with poorer health, including cardiovascular, gastrointestinal, immune, and neurological health problems [29]. Given that Roma mothers are the primary caretakers of children, who are often raised in resource poor environments, their children’s health may be an important correlate of Roma women’s self-reported health.

In addition, stature and body mass were collected using a standard procedure [30]. We included height as an additional measure, as a marker of early-life circumstances and a reliable indicator of fecundity or the maternal ability to reproduce successfully [31,32].

Our final sample included 468 Roma women, who ranged from 16 to 80 years of age. We used cross-sectional measures that count the number of offspring alive at the time of the interview, but controlled for the mother’s age [33,34].

We used descriptive statistics and Chi-square and *t*-tests to detect differences in demographic and reproductive variables between Roma women based on their health status (good health vs. poor health). In addition, we conducted hierarchical logistic regression. Binary variables were coded as dummy variables (1 = presence, 0 = absence), while SES was the only multinominal variable (with three modalities: poor SES, average SES, and above average SES), coded as three dummy variables. In order to avoid problems with expected frequencies of less than five in the statistical analyses, the categories for the children’s health variable were collapsed into two: having a sick child or not having a sick child.

Our goal was to determine whether reproductive history variables appear as predictors of self-rated poor health (presence of chronic disease) among Roma women, correcting for the influence of several independent variables previously shown to influence health status [35,36,37], such as age (continuous variable), lifestyle (smoking 0 = no, 1 = yes; and drinking 0 = no, 1 = yes), poor SES (0 = no, 1 = yes), average SES (0 = no, 1 = yes), above average SES (0 = no, 1 = yes), height (continuous variable), body mass index (BMI, continuous variable), and years of school attendance (continuous variable). Thus, the dependent variable was self-rated poor health/presence of chronic disease (1 = yes, 0 = no) and the independent variables were age at first reproduction (AFR, continuous variable), age at last reproduction (ALR, continuous variable), number of pregnancies (continuous variable), number of surviving children (continuous variable), lifetime duration of breastfeeding (in months, continuous variable), birth spacing (continuous variable), and the health status of one’s children (0 = good health, 1 = poor health).

In the first step, the control variables: smoking, drinking, SES, height, BMI, years of attending school, and age were entered in the model. The second step involved the inclusion of the reproductive history variables.

## 3. Results

The socio-demographic characteristics and health and reproductive variables of the participants in the study are summarized in Table 1.

Roma women were middle-aged, with little schooling and average SES values. The majority were active smokers and within the normal range of BMI. First reproduction was relatively early, with subsequent births every two years, while breastfeeding, on average, was 13.72 months (standard deviation (SD) = 5.91) per child. Duration of the reproductive period was nine years, with the age at last reproduction at 26 years for the entire sample. For women over 40 years of age (who likely, based on Roma reproductive pattern, had finished reproduction), the age at last reproduction was 27.44 (SD = 5.20), while the number of surviving children was 3.91 (SD = 1.88); 75% of women were menopausal, experiencing it at an average age of 46 (SD = 3.03).

Slightly over one third of Roma women (35%) reported poor health, with the most common complaints being hypertension and diabetes. Roma women who reported good health tended to be younger, to have completed more years of elementary schooling, and fell within the scope of a normal BMI range. Roma women in poor health tended to be overweight with limited schooling. The two groups differed on almost all of the reproductive variables except age at first reproduction and birth spacing. Roma women in poor health had longer reproductive periods, more pregnancies, and more extensive breastfeeding, but also more surviving children. There was no difference in rating their children’s health status, SES, or lifestyle (smoking and drinking). Also, there was no difference in height.

Table 2 and Table 3 show the results of the regression models. Table 2 shows the models with controlled variables (Step 1, model 1) and independent variables (Step 2, model 2), while Table 3 shows the exponentiation of the B coefficient* Exp (B) and significance for each variable.

Both models were statistically significant, implying that both the control and independent variables explained the dependent variable, i.e., self-reported poor health among Roma women. Control variables explained 36% of variance of the dependent variable; when independent variables were entered, this percentage rose to 39%.

After correcting for the influence of the control variables, independent variables that explained self-reported poor health among Roma women were the lifetime duration of breastfeeding and number of surviving children. An increase in the lifetime duration of breastfeeding of one month increased the risk of chronic disease occurrence (self-reported poor health) by 1.01 times (odds ratio (OR) = 1.01; 95% confidence of interval (CI) = 1.00–1.02; *p* = 0.044). The number of surviving children also contributed to Roma women’s SR poor health: among women in good health, the average number of surviving children was three, while for women in poor health, this was four (see Table 1). (OR = 1.121; 95% CI = 1.081–1.225; *p* = 0.021).

Of the controlled variables, only BMI showed statistical significance (OR = 1.18; 95% CI = 1.09–1.28; *p* = 0.001). Roma women in poor health had, on average, a higher BMI (Mean = 26.39 ± 3.29) compared to Roma women in good health (Mean = 25.54 ± 2.91). An increase of one unit of BMI raises the possibility of chronic disease occurrence 1.18 times.

## 4. Discussion

To the best of our knowledge, this is the first study that investigated the variation in reproductive investment and its health consequences among Roma women. Although somatic, short-term costs of reproduction were revealed in this population, whilst evolutionary, long-term costs were unobservable—contrariwise, Roma women in poor health contributed more to the gene pool of the next generation than their healthy counterparts.

Thus, in regard to short-term costs, we found that both controlling and independent/reproductive variables were significant in explaining SR poor health (presence of chronic disease) among Roma women. The independent variables that explained self-reported poor health among Roma women were those investments made after birth: lifetime duration of breastfeeding and number of surviving children. A higher BMI also contributed to SR poor health, while no other variable reached statistical significance.

Our findings appear to be consistent with simple trade-off models that suggest inverse relationships between reproductive effort and health. In our study, there was no difference in height among Roma women, and we thus assumed that these women had similar health at the beginning of their reproductive career. Roma mothers invested heavily in their children after birth—by breastfeeding for prolonged periods of time. Cumulative costs of lactation are usually not taken into account by research analyzing the relationships between the fertility, health, and longevity of women, even though the cost of lactation is a very important variable in calculations of total reproductive costs [5]. The age of the baby and the frequency of daily feedings alter the costs of lactation but, on average, lactation requires an additional 626 kcal per day and may last for several years. In our sample the women in poor health spent an average of 4.8 years of their lives breastfeeding, or, in the case of women with 10 surviving children, an average of 14.1 years breastfeeding. In addition, and in line with other studies, we found a significant direct cost associated with childcare, i.e., the number of surviving children also influenced SR poor health among Roma ([38,39,40,41,42,43,44,45,46,47]; but see [19,48,49,50]).

In all studies with a positive correlation between the number of children and physical health, having four or more children was associated with the mothers’ poorer health. In regard to the current sample, Roma women in poor health had an average of four children, while among Roma women in good health, the average number of surviving children was three. Exactly why having four children should produce unfavorable outcomes is not known. Moreover, ethnicity, social context, health, and age of the mother and children influence the degree to which women perceive their children as burdensome [51,52,53,54]. Little attention has been given to understanding parenting stress among low-income, ethnically diverse mothers, but limited results suggest that higher levels of parenting stress and less perceived social support were associated with poor health outcomes. We found no difference in socioeconomic status and children’s characteristics between Roma mothers in regard to their health, but these variables were self-reported and may thus have affected our findings.

We also found that a higher BMI influenced Roma women SR health, but unlike in other studies, height was insignificant [55]. Several prior studies that have reported on the obvious costs of reproduction were conducted among women living in the lower socio-economic strata of a population, or under relatively extreme conditions of malnourishment and disease [11,13,18]. Serbian Roma belong to the lower socio-economic sector of Serbian society but, nonetheless, are well fed [30]. In the current sample, the Roma women fell primarily within the acceptable nutritional status (BMI = 18.5–24.9), although a substantial proportion were overweight and some were obese. There were no underweight women. 

The cost of reproduction needs to be interpreted against the total energy budget [5]. For well-fed women, the number of children is consistently positively related to the risk of obesity, impaired glucose tolerance, non-insulin-dependent diabetes, and cardiovascular diseases [56,57,58]. During each pregnancy, body weight is gained and most women keep at least some of this weight after delivery [59]. Across Europe, poverty significantly increases the probability of being obese and having a higher BMI, but there is still no consensus about causality [60] even though a US-based study found a positive causal link among low income women, federal benefits, and BMI or obesity prevalence [61]. A combination of guaranteed income (intergenerational welfare dependency), unemployment and general physical inactivity, and dietary habits may explain the Roma women’s nutritional status. These may also be reasons why we do not observe any long-term (evolutionary) costs of reproduction in this population: adequate food resources may mean that any long-term costs of reproduction are not sufficiently severe to show up [20,62]. Only one third of Roma women in the current sample reported poor health. Given that many Roma women do not engage in heavy work to obtain the resources necessary to support the family, they may devote energy to reproduction without overly sacrificing their own body condition. 

For humans, adaptation to a given environment is reflected in the number of potentially reproductive offspring produced [63]. Because health is defined in terms of individual functioning and well-being, a condition is often called a disease even if it maximizes fitness. What is regarded as beneficial and harmful depends on observer perspective, genes, individuals, or society [4].

Roma women start reproducing at an optimum age, and continue having children in their most fertile years, thus minimizing potential losses [64,65]. Roma women in poor health have longer reproductive periods than their healthy counterparts, and this provides them with the advantage of greater fertility and more surviving children but also increased body weight. Since chronic diseases are mostly detected at relatively older ages, and given that Roma women finish reproduction in their late twenties, the impact on individual fitness in this study was unobservable. On the other hand, Roma women become grandmothers early, given the universal early age of marriage and reproduction. For instance, in her late thirties, a Roma woman may have several grandchildren; in Serbia, as elsewhere, Gypsy females disperse and in the majority of cases take up patrilocal residence [66]. In our sample, the majority of women lived in a multigenerational extended family consisting of in-laws; older women are more likely to live with their sons and daughters-in-law and are expected to help in childcare. Even if she does get sick later on, it would not affect her descendant- leaving success because the youngest children—her great grandchildren—would be taken care of by their mothers and grandmothers in the prime of their physical health.

The early cessation of reproduction among Roma women is perhaps the most intriguing feature in their reproductive pattern and our finding corresponds to the official census data. In spite of at least a decade of fertile period ahead (menopause is initiated relatively early, at age 46), Roma women and their partners must have made a deliberate decision to stop reproducing after the desired number of children was reached. What probably shaped the human female reproductive schedule is the selection made to minimize reproductive competition between generations within the same social unit [67,68], which is consistent with the Roma residential pattern and reproductive behavior. In addition, after the Serbian Law on Social Welfare was passed, only the first four children in a family are entitled to social benefits, which, interestingly, is exactly the same as the average number of children for women in the present sample. 

Thus, reproduction is adjusted to maternal condition rather than vice versa, and in the case of Roma women, subsidies received from social benefits may help to maintain fertility, feed their children, and balance the costs to maternal health and nutrition that mothers would otherwise face if only relying on their own work efforts [13].

## 5. Conclusions

The results of studies testing relationships between reproduction and health and reproduction and life span are inconsistent, probably due to methodological and theoretical problems (review in [14]). Among Roma women, we found evidence for short-term costs of reproductive investment after birth (duration of breastfeeding, number of surviving children, and higher BMI all contributed to poor health). Consequently, personal sacrifice—breastfeeding and childcare, even though poor health is an outcome—seems crucial for greater reproductive success, i.e., leaving more descendants.

On the other hand, there is no evidence for long-term costs: Roma women who made a relatively larger investment (those with four and more children with a later age at last birth and extensive breastfeeding) had more surviving children. In the long run, received benefits may have helped to increase the Roma mothers’ ability to resist the stress of reproductive investments.

Finally, while our study presents interesting and novel findings on Roma women’s health, there are limitations. In addition to the possibility of biases of self-reported health and other variables, we did not have any detailed descriptions of family relationships and interactions that may influence childcare costs among Roma women. For instance, women who did not have any additional help likely spent more time and energy providing for each additional child. Also, simply adding up the number of children to estimate the energetic and metabolic costs may not be an adequate measure since, for instance, it could be more physiologically demanding to bear and raise boys than girls [69]. Furthermore, parenting style may also influence the degree to which mothers perceive their children as burdensome, dependent on different stages in the life cycle or simply expectations for how children should behave and what mothers are supposed to do for their children; for instance, mothers with more traditional values are more likely to exhibit more frequent conflict with their children which is a major source of parenting stress and may thus contribute to poorer health [70]. Thus, future studies on Roma health should consider personal values as well as other features of Roma traditional life, such as the interaction between Roma grandmothers and their grandchildren, and whether these interactions have any costs and benefits for the wellbeing of Roma women themselves. 

## Figures and Tables

**Table 1:** Socio-demographic characteristics and health and reproductive variables of Roma women.

	SR Good Health/Absence of Chronic Disease *N* = 302	SR Poor Health/Presence of Chronic Disease *N* = 166	Sig.	Total *N* = 468
Children’s health, *n* (%)			0.888 ^a^	
Poor	200 (66.2)	111 (66.9)		311 (66.5)
Good	102 (33.8)	55 (33.1)		157 (33.5)
Smoking, *n* (%)			0.721 ^a^	
No	81 (26.8)	42 (25.3)		123 (26.3)
Yes	221 (73.2)	124 (74.7)		345 (73.7)
Drinking, *n* (%)			0.495 ^a^	
No	241 (79.8)	128 (77.1)		369 (78.7)
yes	61 (20.2)	38 (22.9)		99 (21.2)
SES, *n* (%)			0.210 ^a^	
Poor	126 (41.7)	61 (36.7)		187 (40.0)
Average	159 (52.6)	89 (53.6)		248 (53.0)
Above average	17 (5.6)	16 (9.6)		33 (7.1)
AFR, mean (SD)	17.57 (2.47)	17.22 (2.02)	0.114 ^b^	17.45 (2.33)
ALR, mean (SD)	25.88 (4.80)	27.46 (5.32)	0.03 ^b^	26.46 (5.05)
Number of pregnancies, mean (SD)	5.61 (3.59)	6.72 (3.71)	0.02 ^b^	6.00 (3.67)
Number of surviving children, mean (SD)	3.38 (1.77)	3.84 (1.89)	0.04 ^b^	3.54 (1.83)
Lifetime breastfeeding, mean (SD)	43.84 (31.84)	57.78 (45.62)	0.04 ^b^	48.79 (37.86)
Birth spacing, mean (SD)	2.28 (1.45)	2.33 (1.16)	0.677 ^b^	2.30 (1.36)
height, mean (SD)	160.35 (5.40)	159.69 (5.11)	0.197 ^b^	160.11 (5.30)
BMI, mean (SD)	25.54 (2.91)	26.39 (3.29)	0.000 ^b^	25.20 (3.17)
BMI, *n* (%)			0.000 ^a^	
Underweight (<18.5)	0 (0)	0 (0)		0 (0)
Normal (18.5–24.9)	184 (60.9)	54 (32.5)		238 (50.9)
Overweight (25–30)	101 (33.4)	84 (50.6)		185 (39.5)
Obese (>30)	17 (5.6)	28 (16.9)		45 (9.6)
Years of attending school, mean (SD)	5.21 (3.50)	3.37 (3.03)	0.03 ^b^	4.56 (3.45)
Age, mean (SD)	38.58 (12.57)	52.71 (12.30)	0.02 ^b^	43.59 (14.18)

*n*: number of observation; SD: standard deviation; Sig.: signification; ^a^: Chi-square test was performed; ^b^: *t*-test was performed; SR: self reported; SES: socioeconomic status; AFR: age at first reproduction; ALR: age at last reproduction; BMI: body mass index.

**Table 2:** Model summary.

		Chi-Square	df	Sig.	Nagelkerke R Square
Step 1	Model	138.298	8	0.000	0.362
Step 2	Model	151.880	15	0.000	0.392

df: degrees of freedom.

**Table 3:** Variables in the equation.

	Sig.	Exp (B)	95% CI for Exp (B)	
			Lower	Upper
Smoking	0.066	1.722	0.965	3.073
Drinking	0.845	1.061	0.584	1.929
SES poor	0.409			
SES average	0.182	0.532	0.211	1.343
SES above average	0.269	0.599	0.241	1.487
Height	0.403	1.019	0.974	1.066
BMI	***0.000***	1.185	1.096	1.280
School	0.804	0.988	0.900	1.085
Age	0.061	1.091	1.067	1.117
AFR	0.091	0.900	0.796	1.017
ALR	0.036	1.086	1.005	1.172
Number of pregnancies	0.944	1.003	0.921	1.093
Number of surviving children	***0.021***	1.121	1.081	1.225
Lifetime breastfeeding	***0.042***	1.010	1.000	1.019
Birth spacing	0.176	0.861	0.693	1.070
Children’s health	0.120	0.664	0.396	1.113

CI: confidence of interval; Exp (B): the exponentiation of the B coefficient.
